# Tillage Combined with Straw Return Optimizes Soil Nutrients by Regulating Soil Microbial Properties in Northeast China

**DOI:** 10.3390/plants15071037

**Published:** 2026-03-27

**Authors:** Ping Tian, Meikang Wu, Ming Gao, Pengxiang Sui, Nan Mei, Hua Qi, Zhihai Wu

**Affiliations:** 1 College of Agronomy, Jilin Agricultural University, Changchun 130118, China; tianping@jlau.edu.cn (P.T.); wumeikang@mails.jlau.edu.cn (M.W.); gm876556537@gmail.com (M.G.); meinan413@jlau.edu.cn (N.M.); 2 College of Agronomy, Shenyang Agricultural University, Shenyang 110866, China; suipengxiang1990@163.com (P.S.); qihua10@syau.edu.cn (H.Q.); 3 Institute of Agricultural Resource and Environment, Jilin Academy of Agricultural Sciences (Northeast Agricultural Research Center of China), Key Laboratory of Crop Ecophysiology and Farming System in Northeast China, Ministry of Agriculture and Rural Affairs, Changchun 130033, China

**Keywords:** tillage depth, straw returning, soil nutrients, soil enzyme activity, soil microbial community

## Abstract

The integration of appropriate tillage practices with straw returning can effectively mitigate soil degradation in Northeast China. However, limited research has explored the impacts of different tillage practices combined with varying straw incorporation depths on the structure and diversity of soil microbial communities. In 2016, a field experiment was initiated using a two-factor split-plot design, featuring six treatments: two tillage depths of 10 cm (D10) and 30 cm (D30) combined with three straw management practices—straw mixing incorporation (SM), straw deep burial (SB), and straw removal (SR). Soil samples collected in 2019 were analyzed for multiple soil properties and microbial indices. Results indicated that both straw returning and tillage depth significantly influenced soil organic carbon (SOC), soil total nitrogen (STN), total phosphorus (TP), and total potassium (TK), with the D30 treatment combined with straw returning optimizing soil nutrient contents most effectively. Under straw returning, D10 significantly increased urease activity in the 0–10 cm soil layer, whereas D30 enhanced this enzyme activity in the 10–30 cm soil layer, while β-glucosidase activity was less responsive to tillage depth. For the D10 treatment with straw returning, acid phosphatase activity was markedly higher than that in the straw removal treatment, whereas N-acetyl-β-D-glucosaminidase activity exhibited the opposite trend. Straw-returning methods had no significant effects on the bacterial and fungal Chao1 indices, while the Shannon index was positively correlated with key soil properties. Redundancy analysis (RDA) of microbial community composition at the phylum level and soil environmental factors revealed that soil nutrients in the 0–10 cm soil layer were positively correlated with Actinobacteriota, Ascomycota, and Basidiomycota, whereas the explanatory power of soil nutrients for microbial community variation decreased in the 10–30 cm soil layer. Our results highlight that tillage depth and straw returning can regulate soil microbial community composition and enhance soil nutrient cycling, thereby providing a theoretical basis for optimizing the combined application mode of tillage and straw-returning practices in Northeast China.

## 1. Introduction

The Northeast China Plain, the fourth-largest black soil region in the world, is pivotal for safeguarding China’s food security [1]. In recent years, intensive agricultural practices have led to soil degradation [2,3]. In Northeast China, long-term conventional tillage practices typified by plowing have reduced soil organic matter of the highly fertile black soil, with the black soil layer undergoing rapid erosion at an annual rate of 1.25 mm [4]. The structure and function of the plow layer have declined significantly, threatening soil quality and sustainable utilization [5]. Conservation tillage practices, such as straw returning to the field, can mitigate soil erosion, enhance soil carbon sequestration, and improve soil quality, thus being recommended as alternative soil management measures [6,7]. Nevertheless, while conservation tillage helps preserve soil quality, it may lead to reduced crop yields in semi-humid regions, where soil properties exert a weaker control on yield variation than in semi-arid areas, with the explanatory power ranging from 63% in semi-humid regions to 77–81% in semi-arid regions [8]. Therefore, exploring suitable integrated measures of tillage and straw returning to the field is vital for improving soil quality and promoting the sustainable development of agriculture.

Compared with soils under conventional management, those under organic management develop a more stable and resilient microbial community [9]. Among organic practices, straw returning to the field is recognized as one of the most direct, economical and efficient measures. As a byproduct of crops, straw is rich in organic matter, including nitrogen, phosphorus, potassium and trace elements, and thus plays a vital role in the sustainable development of agriculture [10]. Straw returning to the field increases soil organic carbon storage, elevates the contents of soil total nitrogen and total phosphorus, optimizes soil physicochemical properties, and alters the composition and activity of soil microbial communities [11,12]. In the wheat-maize rotation system, straw returning to the field increases bacterial diversity and elevates the abundance of functional genes associated with carbon, nitrogen, and phosphorus cycling, in comparison with straw removal [13]. However, discrepancies exist among different straw-returning methods: straw crushing and returning to the field can facilitate soil nutrient cycling through straw decomposition, whereas straw mulching and returning to the field improves the economic value and nutrient use efficiency of straw yet has a low nutrient conversion efficiency [14]. Consequently, different straw-returning methods exert distinct impacts on soil physicochemical and microbial properties.

Soil microbial communities are of crucial importance to straw degradation, and the straw degradation process depends largely on the nutrient contents in soil as well as the capacity of soil microorganisms to utilize these nutrients efficiently. Moreover, variations exist in the effects of different soil nutrient contents on the compositions of soil bacteria and fungi [15]. Except for potassium, which exists in a free state in straw and can be directly absorbed, other nutrients need to be decomposed and mineralized by microorganisms before their release [16]. In paddy fields, straw returning to the field under water-saving irrigation regimes significantly increased the contents of soil total nitrogen and organic carbon, enhanced the metabolic pathways of soil microorganisms associated with carbon and nitrogen cycling, and facilitated the migration of straw-derived nutrients to the soil, thereby improving soil quality [17]. Compared with the reduced straw returning treatment, full straw returning significantly increased the carbon use efficiency of bacterial microorganisms and the abundance of fungal microorganisms in the soil [18]. Soil tillage practices can improve soil properties and facilitate the formation of a more complex soil micro-food web structure [19]. For conventional rain-fed farmlands, physical subsoiling is essential yet insufficient to completely address the compaction issue of the plow pan in soil [20]. Therefore, it is imperative to integrate biological amelioration methods to achieve the goal of long-term soil fertility improvement. Tillage and straw returning can alter the physicochemical properties and environmental conditions of soil, and such changes in soil properties directly affect the activities of enzymes associated with soil nutrient transformation [21]. Our previous research findings have confirmed that soil tillage depth directly affects changes in the physicochemical properties of the corresponding soil layers due to the different existing forms of straw in the soil [22]. Variations in soil nutrient contents are directly associated with nutrient release from straw [23], and a positive correlation exists between the stability of soil microbial communities and soil nutrients [24]. Combining tillage practices with different straw returning depths enhances soil nitrogen availability and root nitrogen metabolism, and maize yields can be further increased when a 30 cm tillage depth is adopted under straw mixed incorporation conditions and a 50 cm tillage depth is applied under straw plowing and burial conditions [25]. Therefore, clarifying the key synergistic effects of tillage depth and straw returning to the field can facilitate the sustainable and high-yield cultivation of maize in Northeast China.

Based on the above considerations, we conducted a 4-year field positioning experiment in a continuous maize cropping system to screen appropriate tillage and straw returning modes for improving degraded brown soil. We analyzed soil microbial communities under different tillage depths and straw returning regimes using high-throughput sequencing technology (16S rRNA and ITS) and determined soil nutrient contents and enzyme activities as key evaluation indicators. The objectives of this study were to (1) evaluate the effects of different tillage depths and straw-returning practices on soil physicochemical properties, enzyme activities, and microbial community composition and (2) identify the correlations between soil physicochemical properties and microbial community structure and clarify the key factors regulating these relationships under contrasting straw returning conditions. This study will further elucidate the mechanisms by which tillage and straw returning influence the soil environment, provide a scientific basis for rational tillage and straw management in degraded brown soil, and support food security and environmental sustainability.

## 2. Results

### 2.1. Soil Properties

**Figure 1:**
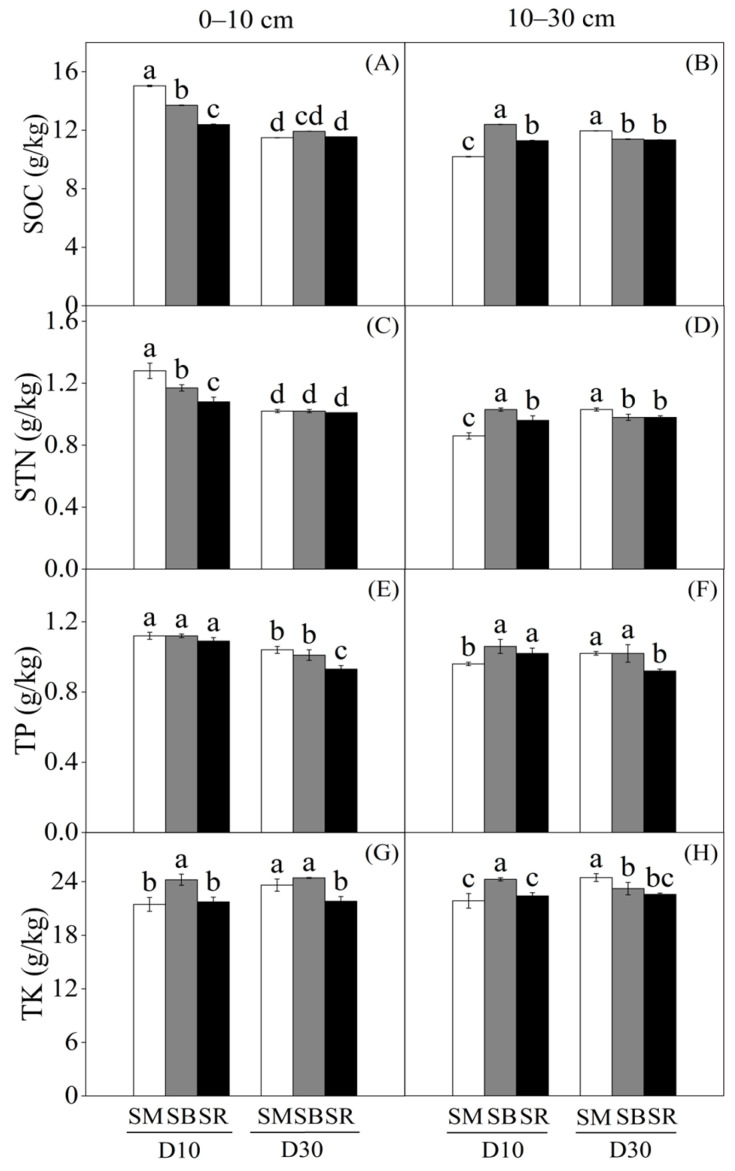
Effects of tillage depth and straw returning on soil organic carbon (SOC), soil total nitrogen (STN), total phosphorus (TP), and total potassium (TK) at 0–10 cm and 10–30 cm soil layers (*n* = 3). SM: Straw mixing incorporation; SB: Straw deep burial; SR: Straw removal; D10: Tillage depth 10 cm; D30: Tillage depth 30 cm. (**A**) SOC at 0–10 cm; (**B**) SOC at 10–30 cm; (**C**) STN at 0–10 cm; (**D**) STN at 10–30 cm; (**E**) TP at 0–10 cm; (**F**) TP at 10–30 cm; (**G**) TK at 0–10 cm; (**H**) TK at 10–30 cm. Different letters in the same soil depth indicate significant differences at *p* < 0.05.

Straw returning and tillage depth significantly affected soil organic carbon (SOC), soil total nitrogen (STN), soil total phosphorus (TP) and soil total potassium (TK) (Figure 1). SOC and STN followed a similar trend, with a significant positive correlation (*p* < 0.01, R^2^ = 0.8116). In the 0–10 cm soil layer, straw returning (SM and SB) significantly increased SOC (Figure 1A) by 21.31% and 10.57% and STN (Figure 1C) by 18.52% and 8.33%, respectively, compared with straw removal (SR) under the 10 cm tillage depth (D10), whereas no significant differences were observed under the 30 cm tillage depth (D30). In the 10–30 cm soil layer, SB under D10 significantly increased SOC content by 21.69% and 9.93% and STN content by 19.77% and 7.29%, respectively, relative to SM and SR under the same tillage depth. In contrast, under D30, SM significantly increased SOC content by 5.47% and STN content by 5.10% compared with SB. No significant differences in SOC (Figure 1B) and STN (Figure 1D) were observed between the two tillage depths for the SR treatment. In the 0–10 cm soil layer, D30 significantly increased TP content under the SM and SB treatments by 11.83% and 8.60% compared with SR, whereas no significant differences were observed among the three straw management methods under D10 (Figure 1E). In the 10–30 cm soil layer, SB significantly increased TP content by 10.42% compared with SM D10 and by 10.87% compared with SR D30 (Figure 1F). The SB D10 treatment significantly increased TK content in both the 0–10 cm (Figure 1G) and 10–30 cm (Figure 1H) soil layers. Additionally, compared with SR under D30, SM and SB under D30 increased TK content by 8.30% and 11.97% in the 0–10 cm soil layer and by 8.33% and 2.84% in the 10–30 cm soil layer, respectively.

Compared with the straw removal (SR) treatment, straw returning (SM and SB) significantly affected soil pH, soil water content (SWC), ammonium nitrogen (NH_4_^+^-N), nitrate nitrogen (NO_3_^−^-N), available phosphorus (AP), and available potassium (AK) (Table 1). Soil pH, SWC, and NO_3_^−^-N content were lower in the 0–10 cm soil layer than in the 10–30 cm layer, whereas NH_4_^+^-N, AP, and AK contents showed the opposite trend. In the 0–10 cm soil layer, the SM D10 treatment significantly increased most soil chemical properties compared with SR D10 and SR D30. In the 10–30 cm soil layer, SWC was significantly higher under SM D10 and SB D10 than under SR D10 by 8.86% and 1.83%, respectively, and SM D30 was higher than SR D30 by 2.80%. Across the 0–30 cm soil profile, straw returning led to significant increases in AP and AK contents within the same tillage depth, compared with SR.

### 2.2. Soil Enzyme Activities

**Figure 2:**
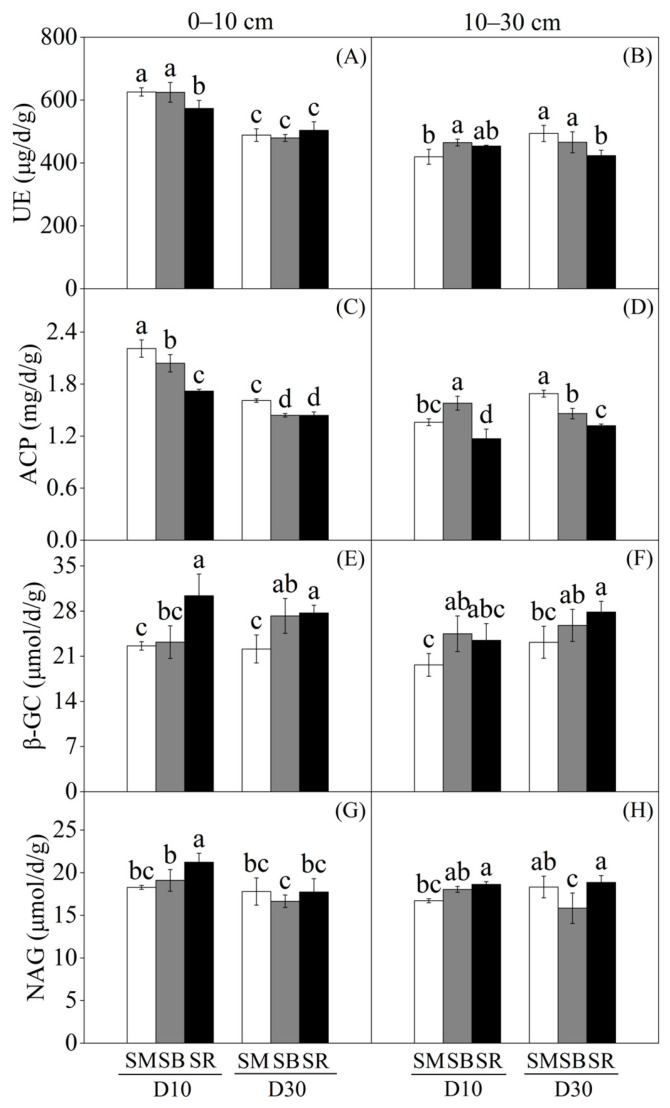
Effects of tillage depth and straw returning on soil enzyme activities at 0–10 cm and 10–30 cm soil layers (*n* = 3). SM: Straw mixing incorporation; SB: Straw deep burial; SR: Straw removal; D10: Tillage depth 10 cm; D30: Tillage depth 30 cm. (**A**) Urease (UE) at 0–10 cm; (**B**) Urease (UE) at 10–30 cm; (**C**) Acid phosphatase (ACP) at 0–10 cm; (**D**) Acid phosphatase (ACP) at 10–30 cm; (**E**) β-Glucosidase (β-GC) at 0–10 cm; (**F**) β-Glucosidase (β-GC) at 10–30 cm; (**G**) N-Acetyl-β-glucosaminidase (NAG) at 0–10 cm; (**H**) N-Acetyl-β-glucosaminidase (NAG) at 10–30 cm. Different letters indicate significant differences among different straw-returning methods of the same soil depth at *p* < 0.05.

Straw incorporation methods and tillage depth exerted significant effects on soil enzyme activities (Figure 2). Soil urease (UE) activity generally decreased with increasing soil depth. In the 0–10 cm soil layer, straw incorporation (SM, SB) significantly increased UE activity by 8.41% and 8.23%, respectively, compared with SR under D10, whereas no significant differences were detected among the three treatments under D30 (Figure 2A). Across all straw incorporation methods, D30 consistently reduced UE activity relative to D10 in this topsoil layer. In the 10–30 cm soil layer, SB significantly increased UE activity by 9.76% compared with SM under D10, while no significant differences were observed under D30 (Figure 2B). Notably, under SM, D30 increased UE activity by 15.03% compared with D10, but this pattern was not observed under SB or SR.

In the 0–10 cm soil layer, straw incorporation (SM, SB) significantly increased acid phosphatase (ACP) activity by 21.95% and 15.55%, respectively, relative to SR under D10. However, under D30, no significant differences were found between SB and SR (Figure 2C). Under all straw incorporation methods, ACP activity was consistently higher under D10 than under D30 in the 0–10 cm soil layer. In the 10–30 cm soil layer, D30 significantly increased ACP activity compared with D10 under SM and SR, but an opposite trend was observed under SB (Figure 2D). This suggests that the response of ACP to tillage depth may be mediated by the straw placement method.

In the 0–10 cm soil layer, β-glucosidase (β-GC) activity followed the order SR > SB > SM under both tillage depths, and tillage depth had no significant effect on β-GC activity under any straw incorporation method (Figure 2E). In the 10–30 cm soil layer under D10, β-GC activity was 19.72% lower in SM than in SB, and under D30, it was 16.81% lower in SM than in SR (Figure 2F). The effects of tillage depth on β-GC activity were consistent with those observed in the topsoil layer (0–10 cm).

In the 0–10 cm soil layer, N-acetyl-β-D-glucosaminidase (NAG) activity tended to be higher in SR than in SM and SB under D10, although no significant differences were detected among treatments (Figure 2G). In the 10–30 cm soil layer under D30, NAG activity was significantly higher in SM and SR than in SB (Figure 2H). This finding indicates that straw burial may suppress NAG activity in deeper soil layers under intensive tillage.

### 2.3. Soil Microbial Abundance

**Figure 3:**
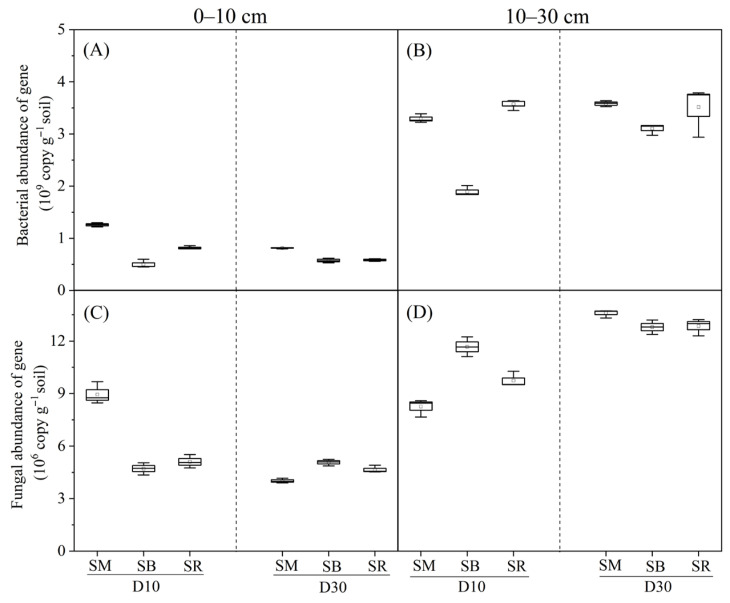
Effects of tillage depth and straw returning on soil bacterial and fungal gene copy numbers at 0–10 cm and 10–30 cm soil layers (*n* = 3). Values are presented as box plots showing the median (circle), interquartile range (box), and minimum and maximum values (whiskers). SM: Straw mixing incorporation; SB: Straw deep burial; SR: Straw removal; D10: Tillage depth 10 cm; D30: Tillage depth 30 cm. (**A**) Bacterial gene abundance at 0–10 cm; (**B**) Bacterial gene abundance at 10–30 cm; (**C**) Fungal gene abundance at 0–10 cm; (**D**) Fungal gene abundance at 10–30 cm.

Bacterial gene copy numbers were significantly lower in the 0–10 cm soil layer than in the 10–30 cm layer (Figure 3A,B). Across the 0–30 cm soil profile, SM consistently maintained higher bacterial gene copy numbers than SB and SR under the same tillage depth, with SB showing the lowest values. In the 0–10 cm soil layer, bacterial gene copy numbers were significantly higher under D10 than under D30 for SM and SR, whereas no significant differences were observed between tillage depths for SB (Figure 3A). In the 10–30 cm layer, the patterns for SM and SR were consistent with those in the topsoil (0–10 cm), while SB showed significantly lower bacterial gene copy numbers under D10 than under D30 (Figure 3B).

Fungal gene copy numbers were significantly higher in the 10–30 cm soil layer than in the 0–10 cm soil layer (Figure 3C,D). In the 0–10 cm layer under D10, SM significantly increased fungal gene copy numbers compared with SB and SR, whereas under D30, SM showed significantly lower values than SB and SR (Figure 3C). Under SM, fungal gene copy numbers were significantly higher under D10 than under D30, while no significant differences were detected between tillage depths for SB or SR. In the 10–30 cm layer under D10, SB significantly increased fungal gene copy numbers compared with SM and SR, whereas under D30, no significant differences were observed among the three straw incorporation methods (Figure 3D). Across all straw incorporation methods, D30 significantly increased fungal gene copy numbers relative to D10.

### 2.4. Bacterial and Fungal Communities Alpha Diversity

In the 0–10 cm soil layer, neither the straw incorporation method nor the tillage depth exerted a significant effect on the bacterial Chao1 index (Table 2). Under the D10 tillage depth, the Simpson index did not differ significantly among the three straw incorporation treatments. In contrast, under D30, the SM treatment yielded a significantly higher Simpson index compared to SB and SR treatments. For the Shannon index, straw incorporation treatments under D10 were associated with significantly higher values than the no-straw control, while no significant differences were detected among SM, SB, and SR under D30. In the 10–30 cm soil layer, the bacterial Chao1 index remained stable between D10 and D30 tillage depths for most straw incorporation methods. The only exception was the SM treatment, where Chao1 was significantly higher under D10 than under D30. The Simpson index showed no significant variation across all treatments in this layer, whereas the Shannon index exhibited significant differences between SM D10 and SM D30.

Across the entire 0–30 cm soil profile, the fungal Chao1 index was not significantly affected by either straw incorporation method or tillage depth. In the 0–10 cm soil layer under D10, both the Simpson and Shannon indices of fungal communities were significantly higher in SM and SR treatments compared to SB. Under D30, however, these indices did not differ significantly among the three straw incorporation methods. When comparing tillage depths within the same straw method, no significant differences in Simpson or Shannon indices were observed for SM and SR. In contrast, under SB, both indices were significantly lower under D10 than under D30. In the 10–30 cm layer under D10, SM and SR treatments again showed significantly higher Simpson and Shannon indices than SB. Under D30, however, the SR treatment exhibited significantly lower values for both indices compared to SM and SB.

**Figure 4:**
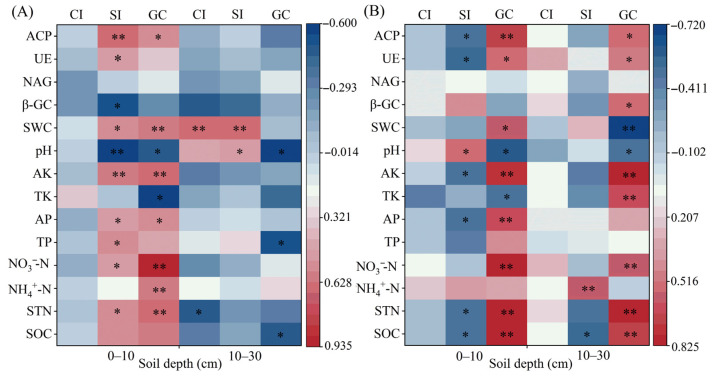
Pearson’s correlation coefficients between soil properties, enzyme activities, microbial α-diversity indices, and microbial gene copy numbers in the 0–30 cm soil layer. (**A**) Bacteria, (**B**) Fungi. Abbreviations: acid phosphatase (ACP), urease (UE), N-acetyl-β-D-glucosidase (NAG), β-glucosidase (β-GC), soil water content (SWC), available potassium (AK), total potassium (TK), available phosphorus (AP), total phosphorus (TP), nitrate (NO_3_^−^-N), ammonium (NH_4_^+^-N), soil total nitrogen (STN), soil organic carbon (SOC), Chao1 index (CI), Shannon index (SI), gene copies (GC). * and ** indicate the correlation is significant at the 0.05 and 0.01 levels, respectively.

In the 0–10 cm soil layer, the bacterial Chao1 index showed no significant correlations with any soil physicochemical properties or enzyme activities (Figure 4A). The Shannon index was significantly and positively correlated with ACP, UE, SWC, AK, AP, TP, NO_3_^−^-N, and STN, while it was significantly and negatively correlated with β-GC and pH. Bacterial gene copy numbers were significantly and positively correlated with ACP, SWC, AK, AP, NO_3_^−^-N, NH_4_^+^-N, and STN, and significantly and negatively correlated with pH and TK. In the 10–30 cm soil layer, SWC was significantly and positively correlated with both the bacterial Chao1 and Shannon indices (*p* < 0.01). pH was significantly and positively correlated with the Shannon index but significantly and negatively correlated with bacterial gene copy numbers. TP and SOC were significantly and negatively correlated with bacterial gene copy numbers, while STN was significantly and negatively correlated with the Chao1 index.

Across the 0–30 cm soil profile, the fungal Chao1 index did not exhibit any significant correlations with soil physicochemical properties or enzyme activities (Figure 4B). In the 0–10 cm soil layer, the fungal Shannon index had a significant negative correlation with ACP, UE, AK, AP, STN, and SOC but was significantly and positively correlated with pH. In the 10–30 cm soil layer, the fungal Shannon index was significantly and positively correlated with NH_4_^+^-N (*p* < 0.01) and had a significant negative correlation with SOC. Fungal gene copy numbers were significantly and positively correlated with ACP, UE, AK, NO_3_^−^-N, STN, and SOC and had a significant negative correlation with pH across the entire 0–30 cm profile.

### 2.5. Soil Microbial Composition

**Figure 5:**
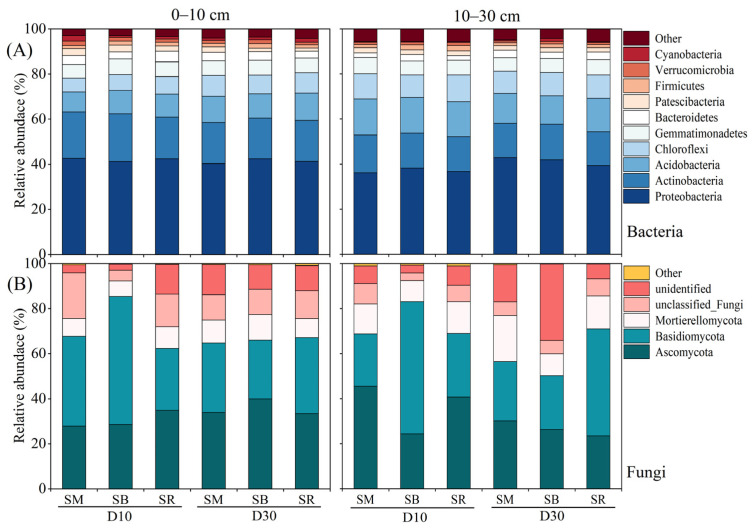
Relative abundance of dominant bacterial and fungal phyla (mean proportion > 1%) in the 0–30 cm soil layer under different tillage and straw returning treatments. SM: Straw mixing incorporation; SB: Straw deep burial; SR: Straw removal; D10: Tillage depth 10 cm; D30: Tillage depth 30 cm. (**A**) Bacterial phyla at 0–30 cm; (**B**) Fungal phyla at 0–30 cm.

Proteobacteria were the most dominant bacterial phylum across both soil layers, accounting for 41.72% (0–10 cm) and 39.19% (10–30 cm) of the community (Figure 5A). Other major phyla included Actinobacteriota for 19.12% and 15.64%, Acidobacteriota for 10.66% and 14.68%, Chloroflexi for 7.90% and 10.61%, Gemmatimonadetes for 6.56% and 6.51%, Bacteroidota for 3.79% and 2.78%, Patescibacteria for 2.26% and 2.06%, Verrucomicrobiota for 1.55% and 1.11%, and Firmicutes for 1.61% and 1.66%. In the 0–10 cm soil layer, D10 significantly increased the relative abundances of Actinobacteriota, Patescibacteria, and Verrucomicrobiota compared with D30, while significantly decreasing those of Acidobacteriota and Chloroflexi. In the 10–30 cm soil layer, D30 was associated with lower relative abundances of Acidobacteriota and Verrucomicrobiota but higher abundances of Proteobacteria and Bacteroidota compared with D10.

Basidiomycota and Ascomycota were the most abundant fungal phyla, with Basidiomycota accounting for 35.81% (0–10 cm) and 34.65% (10–30 cm), and Ascomycota for 33.09% and 31.76%, respectively (Figure 5B). Other notable groups included Mortierellomycota for 9.09% and 13.63%, unclassified Fungi for 12.37% and 6.57%, and undefined fungi for 9.11% and 12.77%. In the 0–10 cm soil layer, D30 significantly increased the relative abundances of Ascomycota and Mortierellomycota, while significantly decreasing those of Basidiomycota and unclassified Fungi. In the 10–30 cm soil layer, D10 significantly increased the relative abundances of Ascomycota and Basidiomycota and decreased that of Mortierellomycota.

**Figure 6:**
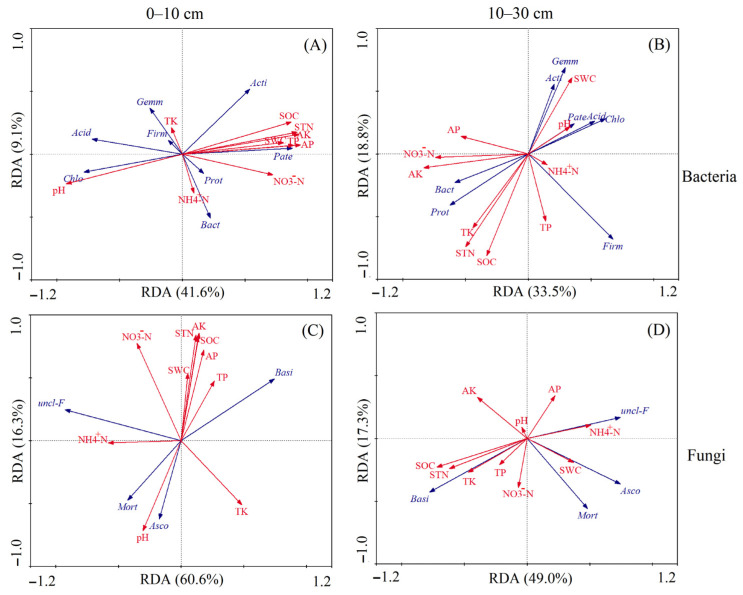
Redundancy analysis (RDA) ordination plots showing the relationships between microbial (bacterial and fungal) phyla (blue arrows) and soil properties (red arrows) in the 0–30 cm soil layer. Panels (**A**,**B**) and (**C**,**D**) show the relationships of soil bacterial and fungal phyla, respectively, with soil properties at the 0–10 cm and 10–30 cm soil depths. The percentages on the axes indicate the variation explained by each RDA axis. Bacterial taxa (blue arrows): Proteobacteria (Prot), Actinobacteria (Acti), Acidobacteria (Acid), Firmicutes (Firm), Bacteroidetes (Bact), Gemmatimonadetes (Gemm), Chloroflexi (Chlo), Patescibacteria (Pate). Fungal taxa (blue arrows): Basidiomycota (Basi), Ascomycota (Asco), unclassified_Fungi (uncl-F), Mortierellomycota (Mort). Soil properties (red arrows): pH, soil water content (SWC), soil organic carbon (SOC), soil total nitrogen (STN), total phosphorus (TP), available phosphorus (AP), total potassium (TK), available potassium (AK), nitrate (NO_3_^−^-N), ammonium (NH_4_^+^-N).

Redundancy analysis (RDA) was used to explore the relationships between dominant microbial phyla and soil physicochemical properties (Figure 6A,B). For bacteria in the 0–10 cm soil layer, the first two RDA axes cumulatively explained 50.7% of the total variation. Nutrient factors such as SOC and STN were positively correlated with Actinobacteriota, while pH was strongly associated with the distribution of Chloroflexi, and NO_3_^−^-N exerts an inhibitory effect on most bacterial taxa. In the 10–30 cm soil layer, the two axes explained 52.3% of the variation. SWC emerged as a core driver for Actinobacteriota and Gemmatimonadetes, pH was more closely linked to Chloroflexi and Acidobacteriota, and the explanatory power of SOC and STN was notably reduced.

For fungi (Figure 6C,D), RDA revealed that in the 0–10 cm soil layer, the first two axes cumulatively explained 76.9% of the variation. Nutrient factors like SOC and STN showed strong positive correlations with Ascomycota and Basidiomycota, NO_3_^−^-N was a key driver for most fungal taxa, and pH was negatively correlated with Mortierellomycota. In the 10–30 cm soil layer, the two axes explained 66.3% of the variation. SWC became the primary driver for Ascomycota and unclassified_Fungi, the association between pH and NH_4_^+^-N was strengthened, and the influence of SOC and STN was significantly diminished.

## 3. Discussion

### 3.1. Effects of Tillage Depth and Straw Returning on Soil Properties

The incorporation of straw rich in nutrients into the soil can enhance root development, thereby improving soil physical properties [26] and increasing the content of organic carbon and available potassium within the plow layer [27]. In the D10 treatment within the 0–10 cm soil layer, the SOC and STN contents in the straw retention treatments were significantly higher than those in the straw removal treatment, whereas no significant differences were observed among treatments under D30 (Figure 1A,C). Our analysis indicated that altered tillage practices resulted in heterogeneous straw distribution across the soil profile, thereby regulating the spatial distribution of SOC and STN. Specifically, straw returning under D10 significantly increased soil organic carbon by 10.57–21.31% and total nitrogen by 8.33–18.52% relative to straw removal in the 0–10 cm soil layer. This aligns with findings that shallow tillage enhances SOC and STN concentrations in the topsoil relative to deep tillage [28]. This difference was likely attributed to stratified straw distribution and stronger microbial immobilization in the topsoil under shallow tillage, whereas deep tillage diluted straw-derived substrates within a larger soil volume, thus weakening the effects on SOC and STN. A higher straw decomposition rate exerts a positive effect on increasing soil nutrient contents [29,30,31] and improves soil fertility [32]. Moreover, tillage combined with straw retention can also affect soil pH, thereby further influencing soil enzyme activities [33].

Compared with the straw removal treatment, straw retention treatments decreased soil pH in the 0–10 cm soil layer (Table 1). The decomposition of straw by microorganisms to release humic acid might be one of the reasons for the reduction in soil pH after straw retention. Tillage practices modify straw decomposition and simultaneously affect straw mineralization and nutrient release [34,35]. Straw retention significantly increased the contents of TP (Figure 1E,F), TK (Figure 1G,H), AP, and AK (Table 1). Phosphorus and potassium are abundant in straw and have a high nutrient release rate during straw decomposition, thereby significantly elevating the contents of soil phosphorus and potassium compared with the straw removal treatment [36]; furthermore, soil mineral nitrogen content is balanced under straw decomposition [37]. Nitrate nitrogen content is the dominant form of nitrogen in dryland farmlands, and it faces a high leaching risk with the implementation of tillage practices. Under the same straw incorporation depth but different incorporation modes, the contents of NO_3_^−^-N and NH_4_^+^-N in the straw return treatment were higher than those in the straw removal treatment (Table 1). Straw retention treatments can increase soil mineral nitrogen content, which is attributed to the activation of key nitrogen cycling genes after straw incorporation [38]. Material cycling and biochemical reactions in soil are directly catalyzed by soil enzymes; thus, enzyme activity determines the transformation rate of soil nutrients and their availability for plant uptake, acts as a key indicator of soil microbial activity in agroecosystems, and promotes soil nutrient cycling [39].

### 3.2. Effects of Tillage Depth and Straw Returning on Soil Enzyme Activities

Optimized farmland management practices can significantly enhance soil enzyme activities by improving soil physical structure and chemical properties [40]. Among these practices, straw incorporation sustains the input of organic carbon sources and nutrient substrates, which in turn elevates the activities of soil enzymes involved in carbon, nitrogen, and phosphorus cycling, thereby boosting ecosystem productivity [38]. Straw mixing incorporation significantly increased the activities of soil urease and acid phosphatase (Figure 2A,C). Further analysis revealed that the higher nitrogen and phosphorus release rates induced by this practice stimulated soil microbial activity, thereby promoting soil enzyme activities—consistent with the findings of previous studies [41,42]. The activities of soil urease and acid phosphatase in the 0-10 cm soil layer were significantly higher than those in the 10–30 cm soil layer (Figure 2A–D), which could be attributed to the higher root length density and stronger nutrient uptake capacity in the subsoil [43]. The intensive absorption of available nutrients by roots further reduced the concentration of substrates involved in enzymatic reactions, which subsequently decreased enzyme synthesis by microorganisms and roots via substrate feedback inhibition, ultimately resulting in lower enzyme activities in the subsoil [22]. Deep tillage enables straw incorporation into the subsoil layer; by introducing organic materials, it not only improves the physicochemical properties of the subsoil but also enhances soil enzyme activities to accelerate the nutrient cycling process [44].

### 3.3. Effects of Tillage Depth and Straw Returning on Soil Microbial Abundance and Composition

Soil microorganisms are the most sensitive component to external environmental factors and are widely used as biological indicators for soil quality assessment. Straw incorporation increases soil organic carbon storage and availability [45]. These changes, in conjunction with alterations in soil nutrient contents and pH, directly affect the structure and activity of soil microbial communities [46]. The gene copy numbers of both bacteria and fungi were significantly higher in the 10–30 cm layer (Figure 3B,D) than in the 0–10 cm topsoil (Figure 3A,C). This difference can be explained by the greater exposure of surface microbes to external environmental factors (e.g., temperature and soil moisture), which limit their abundance in the uppermost layer [47]. Straw mixing incorporation at a 10 cm tillage depth significantly elevated the gene copy numbers of bacteria and fungi in the 0–10 cm soil layer, where microbial gene abundance was significantly correlated with changes in soil physicochemical properties (Figure 4A,B). In the 10–30 cm soil layer, bacterial abundance was less influenced by environmental factors (Figure 4A), whereas fungal abundance exhibited a significant response to such variations (Figure 4B). This pattern indicated that soil fungi were more sensitive to the modes of crop residue return than bacteria, a finding consistent with previous studies [48,49]. The greater sensitivity of fungi can be attributed to their hyphal growth form in soil, which elicits a pronounced response to physical soil disturbances [50]. In the treatments of tillage and straw return, Proteobacteria and Actinobacteriota were the dominant phyla in the bacterial community within the 0–30 cm soil layer (Figure 5A), while Ascomycota and Basidiomycota dominated the fungal community (Figure 5B). This result was consistent with the findings of a previous study on the rice-wheat rotation system, where Ascomycota and Basidiomycota were the dominant taxa in the fungal community [51], highlighting that Ascomycota acts as the primary fungal decomposer in agricultural and other soil ecosystems [52]. Soil microbial taxa at the phylum level exhibited divergent responses to environmental factors under different straw return methods and tillage depth treatments, among which the dominant bacterial and fungal phyla all showed a significant positive correlation with soil organic carbon (Figure 6A–D). These findings suggest that fungal community structure in the topsoil is primarily regulated by nutrient availability, whereas in the subsoil, it is more strongly controlled by soil water content and pH. This reflects the differential effects of vertical soil heterogeneity on the relationships between microbial communities and their environment. This phenomenon is primarily attributed to the fact that soil organic matter acts as an energy source for microbial metabolism, and its content and composition directly modulate microbial community structure [53,54].

## 4. Materials and Methods

### 4.1. Field Site Description

**Figure 7:**
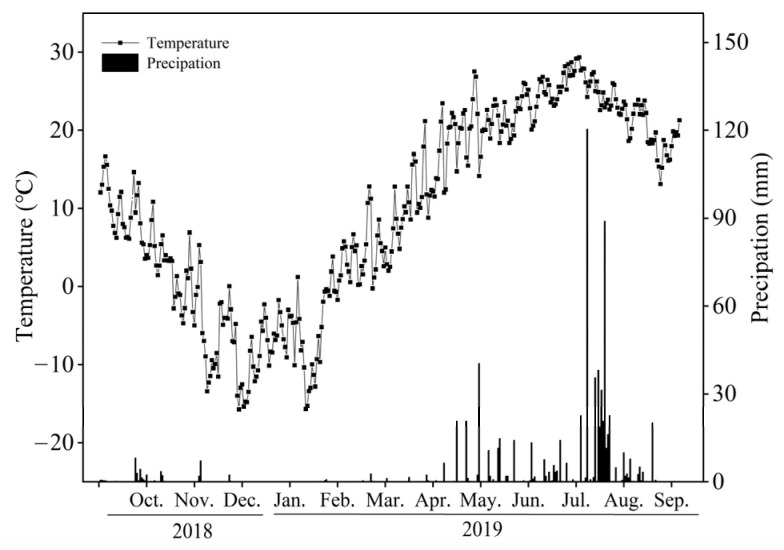
Variations in precipitation and temperature from October 2018 to September 2019.

The field experiment was conducted from 2016 to 2019 at the Experimental Station of Shenyang Agricultural University (41°52′ N, 123°56′ E; 43 m above sea level) in Liaoning province, China. The data and results presented in this study were obtained from October 2018 to September 2019 (a one-year period). This region is characterized by a sub-humid warm-temperate continental climate, with an average annual temperature of 9.17 °C, and an annual precipitation of 714 mm. The soil is classified as a quaternary brown soil (Cambisol, FAO soil classification) with a loam texture. Maize had been grown under a continuous single-cropping system for decades before the experiment was established in 2016. Precipitation served as the sole water source, and no irrigation was applied during the experimental period. Straw returning was implemented before spring sowing in the first year, while in the second to fourth years, straw returning was conducted in autumn after the annual maize harvest. Maize straw was returned to the soil at a rate of 6000 kg ha^−1^, and was crushed into 2–5 cm segments before soil incorporation. At the initiation of the experiment, the basic properties of the topsoil (0–20 cm) were as follows: soil organic carbon (SOC) 10.81 g kg^−1^, soil total nitrogen (STN) 0.92 g kg^−1^, available phosphorus (AP) 51.17 mg kg^−1^, and available potassium (AK) 128.49 mg kg^−1^. During the 2018 and 2019 growing seasons, the daily average temperature was 9.85 °C, and total annual precipitation was 764.4 mm, with the maximum monthly rainfall (438.6 mm) occurring mainly in August (Figure 7). Although some heavy rainfall events were observed, these events were within the normal range of local climate characteristics and did not result in substantial surface runoff or water loss. These climatic conditions were representative of a normal year, consistent with the local long-term average, and thus suitable for regional comparative analysis with other black soil regions. The experiment was arranged in a two-factor split-plot design with three replications. Factor I was straw management, with three levels: straw mixing incorporation (SM), straw deep burial (SB), and straw removal (SR). Factor II was tillage depth, with two levels: 10 cm (D10) and 30 cm (D30). The main plots were assigned to straw management treatments, and the subplots were allocated to tillage depth treatments. SM, SB, and SR represented simulated rotary tillage, moldboard plowing, and no-tillage treatments in the field, respectively. The experimental units were constructed from stainless steel plates (1.5 m × 1.2 m × 0.7 m) with an open bottom. Seed fertilizer was applied at maize sowing in early May, consisting of 75 kg ha^−1^ nitrogen (N) from urea, 90 kg ha^−1^ phosphorus pentoxide (P_2_O_5_) from superphosphate, and 90 kg ha^−1^ potassium oxide (K_2_O) from potassium chloride. In addition, 150 kg ha^−1^ N was top-dressed at the maize jointing stage to satisfy the nutrient requirements during critical crop growth periods. No pesticides or chemical herbicides were applied during the entire experimental period; weeds were removed manually by hand to avoid disturbance to the topsoil layer structure and properties, and no obvious crop pests or diseases were observed.

### 4.2. Soil Sampling and Analysis

Soil sampling and analysis were conducted after maize harvest in 2019. In each plot, three soil cores were collected randomly from 0–10 cm and 10–30 cm layers, respectively, using a soil corer with a 5 cm internal diameter. The three soil cores from the same layer in each plot were thoroughly mixed to form one composite sample. The fresh soil samples were divided into two subsamples. One subsample was air-dried, ground, and passed through 20-mesh and 100-mesh sieves for analysis of soil chemical properties. The other subsample was sieved through a 2 mm sieve in the field, then stored at 4 °C and −80 °C for subsequent determination of soil enzyme activities and microbial community composition.

Soil pH was determined at a soil-to-water ratio of 1:2.5 (*w*/*v*) using a digital pH meter (PHSJ-3F, Leici, Shanghai, China). Soil water content (SWC) was measured by oven drying at 105 °C to a constant mass. Nitrate nitrogen (NO_3_^−^–N) and ammonium nitrogen (NH_4_^+^–N) were extracted with 2 M KCl for 1 h, and their concentrations were determined using an auto-discrete analyzer (SmartChem 200, AMS Alliance, Paris, France) [55]. Available phosphorus (AP) was measured by the sodium bicarbonate Olsen method, and available potassium (AK) was determined by flame photometry. Soil organic carbon (SOC) and soil total nitrogen (STN) were analyzed using an elemental analyzer (EA 3000, Eurovector, Pavia, Italy). Total phosphorus (TP) and total potassium (TK) were determined by digesting samples followed by spectrophotometry and flame photometry, respectively. Soil enzyme activities were determined using commercial reagent kits (Suzhou Keming, Suzhou, China). The measurement principles are briefly described as follows: Soil urease (UE) activity was determined using the indophenol blue colorimetric method, based on the amount of ammonium nitrogen produced via urea hydrolysis. Soil β-glucosidase (β-GC) activity was assayed by measuring p-nitrophenol released from the catalytic hydrolysis of p-nitrophenyl-β-D-glucopyranoside, with absorbance detected at 400 nm. Soil acid phosphatase (ACP) activity was determined by quantifying phenol released from the catalytic hydrolysis of disodium phenyl phosphate. Soil N-acetyl-β-D-glucosaminidase (NAG) activity was measured by detecting p-nitrophenol produced from the decomposition of p-nitrophenyl-N-acetyl-β-D-glucosaminide, with absorbance recorded at 400 nm.

### 4.3. DNA Extraction and Real-Time PCR

Soil microbial DNA was extracted from 0.3 g of frozen soil using the PowerSoil DNA Isolation Kit (MOBIO Laboratories, Carlsbad, CA, USA) following the manufacturer’s instructions. The quality of extracted DNA was examined by electrophoresis on a 1.2% agarose gel, and DNA concentration was determined using a NanoDrop 2000 Spectrophotometer (Thermo Scientific, Wilmington, DE, USA). The V3–V4 hypervariable region of the bacterial 16S rRNA gene was amplified with the primer pair 338F (5′–ACTCCTACGGGAGGCAGCA–3′) and 806R (5′–GGACTACHVGGGTWTCTAAT–3′) [56]. The fungal internal transcribed spacer (ITS1) region was amplified using the primers ITS5F (5′–GGAAGTAAAAGTCGTAACAAGG–3′) and ITS2R (5′–GCTGCGTTCTTCATCGATGC–3′) [57].

PCR amplification was performed in a reaction mixture containing 5 μL of 5× reaction buffer, 5 μL of 5× GC buffer, 2 μL of dNTP (2.5 mmol L^−1^), 1 μL of forward primer (10 μmol L^−1^), 1 μL of reverse primer (10 μmol L^−1^), 1 μL of DNA template, 9.75 μL of ddH_2_O, and 0.25 μL of Q5 DNA polymerase (New England Biolabs, Beijing, China). The thermal cycling conditions were set as follows: initial denaturation at 98 °C for 5 min; 25 cycles for bacteria and 28 cycles for fungi, with each cycle consisting of denaturation at 98 °C for 30 s, annealing at 52 °C for 30 s, and extension at 72 °C for 30 s; followed by a final extension at 72 °C for 5 min. PCR products targeting the bacterial 16S rRNA V3–V4 region and fungal ITS1 region were recovered from a 2% agarose gel and used to construct sequencing libraries for Illumina MiSeq sequencing.

Operational taxonomic units (OTUs) were clustered at a 97% similarity threshold using QIIME software (Version 1.17). Alpha-diversity indices, including the Shannon and Simpson indices, and the Chao1 richness estimator were calculated to characterize the diversity and richness of bacterial and fungal communities.

The abundances of bacterial 16S rRNA and fungal ITS1 genes were quantified by real-time quantitative PCR (qPCR) using a CFX96 system (Bio-Rad, Hercules, CA, USA) and AceQ qPCR SYBR Green Master Mix (Wuhan Jianbo Yahan Biotechnology, Wuhan, China). Each reaction mixture contained 7.5 μL of 2× SYBR Green Mix, 1 μL of DNA template, and corresponding primers. The qPCR program consisted of an initial denaturation at 95 °C for 5 min, followed by 40 cycles of 95 °C for 10 s, 55 °C for 15 s, and 75 °C for 30 s, and a melting curve program of 95 °C for 15 s, 60 °C for 60 s, and 95 °C for 45 s. Cycle threshold (Ct) values were analyzed using Bio-Rad CFX Manager software (ver. 3.1, Bio-Rad, Hercules, CA, USA).

### 4.4. Statistical Analysis

Differences in soil properties, enzyme activities, alpha-diversity indices, and microbial abundances, as affected by tillage depth, straw management, and their interaction, were analyzed using two-way analysis of variance (ANOVA) with SPSS 23.0 (SPSS Inc., Chicago, IL, USA). Duncan’s multiple range test was used to separate treatment means at the 0.05 probability level (*p* < 0.05). Relationships between soil properties and microbial community composition were assessed using redundancy analysis (RDA) in CANOCO 4.5 (Microcomputer Power, Ithaca, NY, USA). All Figures were generated using Origin 2021 (Originlab, Northampton, MA, USA).

## 5. Conclusions

Our results demonstrated that tillage depth and straw returning can significantly regulate soil nutrient status, enzyme activities, and microbial community structure in the investigated agricultural region of Northeast China. Combined with straw returning, deep tillage (D30) exerted a superior effect on improving soil nutrients, including SOC and STN. The interaction between tillage depth and straw returning had a significant regulatory effect on urease activity, whereas β-glucosidase activity was less directly affected by tillage depth. Straw returning supplied substrates for acid phosphatase in the plow layer, but it inhibited the activity of N-acetyl-β-D-glucosaminidase. The Shannon index was closely correlated with soil enzyme activities and physicochemical properties and thus can serve as an important microbial indicator for characterizing changes in soil environmental conditions. Notably, from a landscape perspective, the coupling relationship between microbial community composition and soil environmental factors exhibited significant vertical variations across soil layers. Nutrient factors were the core drivers of microbial community structure in the 0–10 cm soil layer, while microbial community structure in the 10–30 cm soil layer was more dependent on soil moisture and pH conditions, highlighting the differential effects of vertical soil heterogeneity on the microbe-environment interactions. These findings confirmed that tillage depth combined with straw returning can optimize soil nutrient cycling by regulating the soil microecological environment and microbial community structure. Therefore, deep tillage (D30) combined with straw returning is recommended to enhance soil carbon sequestration and promote microbial diversity in this region and other areas with similar soil-landscape conditions. Further research should aim to elucidate the long-term impacts of these management practices across different landscape positions on the stability of soil ecological processes and crop productivity under diverse environmental conditions.

## Figures and Tables

**Table 1:** Soil pH, SWC, NH_4_^+^-N, NO_3_^−^-N, available phosphorus, and available potassium in the 0–30 cm soil layer under different tillage and straw returning treatments.

Soil Depth (cm)	Treatment		pH	Soil Water Content (%)	NH_4_^+^-N (mg kg^−1^)	NO_3_^−^-N (mg kg^−1^)	Available Phosphorus (mg kg^−1^)	Available Potassium (mg kg^−1^)
0–10	SM	D10	5.64 d	15.83 a	6.26 ab	9.35 a	13.31 a	116.99 a
		D30	6.04 b	15.42 ab	6.57 a	5.86 b	8.04 d	88.57 d
	SB	D10	5.69 d	15.61 ab	3.60 d	4.47 c	12.26 b	104.93 b
		D30	6.20 a	15.34 ab	5.72 b	3.15 d	7.66 e	82.07 f
	SR	D10	5.89 c	15.35 ab	4.47 c	5.46 b	11.14 c	95.32 c
		D30	6.21 a	15.15 b	4.09 cd	4.02 c	6.84 f	84.85 e
10–30	SM	D10	6.39 a	17.81 a	3.77 ab	4.91 cd	7.61 b	69.15 d
		D30	6.12 c	16.54 bc	4.33 a	7.02 a	7.96 a	78.70 b
	SB	D10	6.34 a	16.66 b	3.49 bc	5.20 bc	7.37 c	74.85 c
		D30	6.24 b	15.77 e	4.07 ab	4.60 d	8.02 a	82.94 a
	SR	D10	6.13 c	16.36 c	4.22 a	4.03 e	7.02 d	62.82 e
		D30	6.14 c	16.09 d	2.94 c	5.66 b	7.11 d	77.32 bc

Values are means of three replicates. SM: Straw mixing incorporation; SB: Straw deep burial; SR: Straw removal; D10: Tillage depth 10 cm; D30: Tillage depth 30 cm. Different letters indicate significant differences among different straw-returning methods of the same soil depth at *p* < 0.05.

**Table 2:** Alpha diversity indices of soil bacteria and fungi in the 0–30 cm soil layer under different tillage and straw returning treatments.

Soil Depth (cm)	Treatment		Bacteria			Fungi		
			Chao1	Simpson	Shannon	Chao1	Simpson	Shannon
0–10	SM	D10	4601.89 ns	1.00 a	10.91 a	332.61 ns	0.88 a	5.23 a
		D30	4732.63 ns	1.00 a	10.78 ab	333.07 ns	0.93 a	5.69 a
	SB	D10	4837.87 ns	1.00 a	10.90 a	283.78 ns	0.75 b	4.27 b
		D30	4743.46 ns	0.99 b	10.54 b	320.24 ns	0.95 a	5.96 a
	SR	D10	4252.90 ns	1.00 a	10.53 b	362.30 ns	0.95 a	6.05 a
		D30	4641.01 ns	0.99 b	10.54 b	344.07 ns	0.90 a	5.66 a
10–30	SM	D10	4946.79 a	1.00 ns	10.90 a	303.08 ns	0.97 a	6.39 a
		D30	4337.70 b	1.00 ns	10.56 abc	365.95 ns	0.94 a	5.91 a
	SB	D10	4602.75 ab	1.00 ns	10.84 ab	292.19 ns	0.77 b	4.43 c
		D30	4381.45 b	0.99 ns	10.48 bc	310.87 ns	0.94 a	5.73 ab
	SR	D10	4527.05 ab	0.99 ns	10.57 abc	318.02 ns	0.97 a	6.57 a
		D30	4091.05 b	0.99 ns	10.37 c	339.08 ns	0.79 b	4.69 bc

SM: Straw mixing incorporation; SB: Straw deep burial; SR: Straw removal; D10: Tillage depth 10 cm; D30: Tillage depth 30 cm. Different letters in the same column indicate significant differences at *p* < 0.05 in the same soil depth; “ns” indicates no significant differences.

## Data Availability

Data will be made available on request.
